# Efficient Gene Knockdown in Mouse Oocytes through Peptide Nanoparticle-Mediated SiRNA Transfection

**DOI:** 10.1371/journal.pone.0150462

**Published:** 2016-03-14

**Authors:** Zhen Jin, Ruichao Li, Chunxiang Zhou, Liya Shi, Xiaolan Zhang, Zhixia Yang, Dong Zhang

**Affiliations:** State Key Laboratory of Reproductive Medicine, Nanjing Medical University, Nanjing, Jiangsu, China; Institute of Zoology, Chinese Academy of Sciences, CHINA

## Abstract

The use of mouse oocytes as a model for studying female meiosis is very important in reproductive medicine. Gene knockdown by specific small interfering RNA (siRNA) is usually the first step in the study of the function of a target gene in mouse oocytes during *in vitro* maturation. Traditionally, the only way to introduce siRNA into mouse oocytes is through microinjection, which is certainly less efficient and strenuous than siRNA transfection in somatic cells. Recently, in research using somatic cells, peptide nanoparticle-mediated siRNA transfection has been gaining popularity over liposome nanoparticle-mediated methods because of its high efficiency, low toxicity, good stability, and strong serum compatibility. However, no researchers have yet tried transfecting siRNA into mouse oocytes because of the existence of the protective zona pellucida surrounding the oocyte membrane (vitelline membrane). We therefore tested whether peptide nanoparticles can introduce siRNA into mouse oocytes. In the present study, we showed for the first time that our optimized program can efficiently knock down a target gene with high specificity. Furthermore, we achieved the expected meiotic phenotypes after we knocked down a test unknown target gene TRIM75. We propose that peptide nanoparticles may be superior for preliminary functional studies of unknown genes in mouse oocytes.

## Introduction

Gene knockdown through RNA interference is one of the most popular and powerful tools in cell biology studies. Diverse standardized protocols using double-stranded RNA (500–600 bps) [1], short hairpin RNA (shRNA, 23–29 bps) [2], or small interfering RNA (siRNA, 21–23 bps) [3] are optimized and used for different cell types. Among these, nanoparticle-mediated siRNA transfection is the most widely used. In recent years, multiple genome-wide studies have identified many new candidates involved in certain important biological processes by virtue of this technique [1–3]. Nanoparticles, also known as ultrafine particles, have a diameter of <100 nm. According to their structural composition, nanoparticles are liposome-based [4] or peptide-based [5–6]. Recently, peptide-based nanoparticles have been increasingly replacing liposome-based nanoparticles for introducing siRNA into cells owing to their high efficiency, low toxicity, good stability, and strong serum compatibility [5–6].

The mouse oocyte is a good cell model for female meiosis studies that will eventually contribute to human eugenics. However, less information is known about meiosis than mitosis (in Pubmed, 5.7% vs 15.6% in all cell cycle studies). One unfavorable factor is that it is more difficult and strenuous to collect mouse oocytes; the other major one is that microinjection has to be employed for silencing a target unknown gene through specific siRNA in mouse oocytes [7–8]. This is because the zona pellucida around the oocyte membrane (vitelline membrane) can almost completely block the direct uptake of siRNA, entry of lentivirus-mediated shRNA vector, or transfection of liposome nanoparticle-based siRNA [9]. Until now, no research has tested whether peptide nanoparticle-mediated siRNA transfection can be a feasible tool for gene knockdown in mouse oocytes.

Our laboratory is devoted to establishing a standardized and highly operable method for gene knockdown in mouse oocytes through siRNA transfection instead of microinjection. After many trials with several commercial peptide nanoparticle transfection reagents, we have successfully selected the most appropriate reagent that can knock down genes in a highly efficient manner as well as with very low toxicity [10–11].

## Materials and Methods

### General chemicals & reagents and animals

Chemicals & reagents were obtained from Sigma unless otherwise stated. 3~4 week-old female ICR mice used in this study were from Vitalriver experimental animal technical co., LTD of Beijing. All animal experiments were approved by the Animal Care and Use Committee of Nanjing Medical University (approval No:14030158) and were performed in accordance with institutional guidelines. Prior to oocyte collection (usually 1~3 days), mice were temporarily kept in SPF-level clean room in the animal core facility of Nanjing Medical University.

### Antibodies

Mouse monoclonal anti-β-actin (Cat#: A5316-100) was purchased from Santa Cruz (St. Louis, MO, USA). Rabbit polyclonal anti-trim75 (Cat#: sc-249091) was purchased from Santa Cruz (Dallas, Texas, USA). Mouse monoclonal anti-β-tubulin antibody (Cat#: sc-5274) antibody was purchased from Santa Cruz (Dallas, Texas, USA). Human anti-centromere CREST antibody (Cat#:15–234) was purchased from Antibodies Incorporated (Davis, CA, USA). Cy2-conjugated donkey anti-mouse IgG (Code:715-225-150), rhodamine(TRITC)-conjugated donkey anti-goat IgG (Code:705-025-147), and 647-conjugated donkey anti-Human IgG (Code:709-605-149) were purchased from Jackson ImmunoResearch Laboratory (West Grove, PA, USA). Horseradish Peroxidase (HRP)-conjugated rabbit anti goat IgG and HRP-conjugated goat anti mouse IgG were purchased from Vazyme (Nanjing, jiangsu, China).

### Oocytes collection and culture

For oocyte collection, we always picked female ICR mice in natural estrus instead of applied super ovulation. Usually 20–50 immature oocytes arrested in prophase I (GV oocytes) could be obtained from the ovaries of each mouse. Exact numbers of oocytes used in each experiment were included into corresponding figures. The mice were sacrificed by cervical dislocation and ovaries were isolated and placed in operation medium (Hepes) with 2.5 μM milrinone and 10% fetal bovine serum (FBS) (Gibco). Oocytes were released from the ovary by puncturing the follicles with a hypodermic needle. Cumulus cells were washed off the cumulus-oocyte complexes and every 50 Isolated denuded oocytes were placed in 100-ul droplets of culture medium under mineral oil in plastic dishes (BD). The culture medium was MEM+ (MEM with 0.01mM EDTA, 0.23mM Na-pyruvate,0.2mM pen/sterep, 3mg/ml BSA and 20% FBS). Oocytes were cultured at 37.0°C, 5% O_2,_ 5% CO_2_ in humidified atmosphere. Prior to *in vitro* maturation (IVM), all culture medium include 2.5 μM milrinone to prevent resumption of meiosis. Particularly, two different sorts of Gibco FBS were used in this study: for all siRNA related experiments, we used Performance Plus grade FBS (Cat No: 16000–044; Endotoxin ≤5 EU/ml, Hemoglobin ≤10 mg/dl); for all other experiments, we used Secure grade FBS (Cat No: 16000–044; Endotoxin ≤10 EU/ml, Hemoglobin ≤25 mg/dl) that is being widely used in regular cell culture.

### SiRNA production and transfection

Sequences of all DNA templates for siRNA production are listed in Table 1. The sequence of control templates is a mock sequence that does not specifically bind to any mRNA from the mouse genome. DNA templates against four different coding for DNA sequence (CDS) regions of TRIM75 siRNA were designed online through BLOCK-iT™ RNAi Designer (http://rnaidesigner.invitrogen.com/rnaiexpress/) with some modification. Sequence specificity was verified through a blast homology search.

**Table 1:** DNA oligos for siRNA production.

Target Site	DNA templates
437–461^1^	Oligo1: GGATCCTAATACGACTCACTATAGCAAGCTCCTGAAGTGGGAAGTGAA^2^
437–461^1^	Oligo2:AATTCACTTCCCACTTCAGGAGCTTGCTATAGTGAGTCGTATTAGGATCC^2^
437–461^1^	Oligo3: GGATCCTAATACGACTCACTATATTCACTTCCCACTTCAGGAGCTTGC^2^
437–461^1^	Oligo4:AAGCAAGCTCCTGAAGTGGGAAGTGAATATAGTGAGTCGTATTAGGATCC^2^
631–634^1^	Oligo1: GGATCCTAATACGACTCACTATAGAGAAGAGACTGCTTGATAACATA^2^
631–634^1^	Oligo2:AATATGTTATCAAGCAGTCTCTTCTCTATAGTGAGTCGTATTAGGATCC^2^
631–634^1^	Oligo3: GGATCCTAATACGACTCACTATATATGTTATCAAGCAGTCTCTTCTC^2^
631–634^1^	Oligo4:AAGAGAAGAGACTGCTTGATAACATATATAGTGAGTCGTATTAGGATCC^2^
718–742^1^	Oligo1: GGATCCTAATACGACTCACTATAGAGCTCTCCGAAGCCAAGATGTTGT^2^
718–742^1^	Oligo2:AAACAACATCTTGGCTTCGGAGAGCTCTATAGTGAGTCGTATTAGGATCC^2^
718–742^1^	Oligo3: GGATCCTAATACGACTCACTATAACAACATCTTGGCTTCGGAGAGCTC^2^
718–742^1^	Oligo4:AAGAGCTCTCCGAAGCCAAGATGTTGTTATAGTGAGTCGTATTAGGATCC^2^
880–902^1^	Oligo1: GGATCCTAATACGACTCACTATAGACAACGTCACTCTAGACCTGAA^2^
880–902^1^	Oligo2:AATTCAGGTCTAGAGTGACGTTGTCTATAGTGAGTCGTATTAGGATCC^2^
880–902^1^	Oligo3: GGATCCTAATACGACTCACTATATTCAGGTCTAGAGTGACGTTGTC^2^
880–902^1^	Oligo4:AAGACAACGTCACTCTAGACCTGAATATAGTGAGTCGTATTAGGATCC^2^
Control^3^	Oligo1: GGATCCTAATACGACTCACTATACCTACGCCACCAATTTCGTTT^2^
Control^3^	Oligo2:AAAAACGAAATTGGTGGCGTAGGTATAGTGAGTCGTATTAGGATCC^2^
Control^3^	Oligo3: GGATCCTAATACGACTCACTATAAAACGAAATTGGTGGCGTAGG^2^
Control^3^	Oligo4:AACCTACGCCACCAATTTCGTTTTATAGTGAGTCGTATTAGGATCC^2^

^1^ The numbers are the starting and ending position of the target sites in TRIM75 CDS (NM_001033429.2 in NCBI).

^2^ two pairs of DNA oligos are needed for for each double-stand siRNA. Oligo 2 is complementary with oligo 1 except an "AA" overhang at 5'; Oligo 3 is complementary with oligo 4 except an "AA" overhang at 5'. Anealed Oligo 1 and 2 will be the template for the forward stand of siRNA and anealed Oligo 3 and 4 will be the template for the reverse complementary strand of siRNA. Reactions for the production of forward or reverse complementary single-stand siRNA are set up separately, finally combined together and annealed to generate a stable, ready-to-use double-strand siRNA. In each oligo, gene-specific sequences are underlined, other sequences are for recognition and binding by T7 RNA polymerase.

^3^ Control siRNA does not target to any mRNA sequence in mouse.

SiRNAs were produced using the T7 RiboMAX™ Express RNAi System (Promega) according to the manufacturer’s instructions. Briefly, for each double-stranded siRNA against one of the four TRIM75 CDS regions, two pairs of synthesized complementary single-stranded DNA oligonucleotides were first annealed to form two double-stranded DNA templates. Subsequently, two complementary single-stranded siRNAs were separately synthesized in accordance with these two templates and then annealed to form a final double-stranded siRNA. Next, the siRNA was purified by conventional phenol/chloroform/isoproponal precipitation, which was then aliquoted and stored at −80°C after a quality check on the agarose gel. A ready-to-use siRNA mixture was prepared by mixing siRNAs against four target regions together at an equal molar ratio to a final concentration of 5 μM.

For siRNA transfection, the N-TER^™^ Nanoparticle siRNA Transfection System (Sigma) was used. Briefly, two tubes, one containing 1.1 μl N-TER™ nanoparticles in 5.15 μl nuclease-free water (Acros Organics) and the other containing 1.625 μl of siRNA (5 μM) mixture in 4.625 μl of siRNA dilution buffer (provided by the kit) were set up; they were then gently mixed together and incubated at room temperature (RT) for 20 min. Next, the siRNA–nanoparticle complex solution was added into a 100-μl medium drop containing 50 oocytes. After a 12–14 h treatment, the oocytes were washed to remove the nanoparticle-containing medium. After a period of 1–2 h, another one or two rounds of siRNA treatment was performed, depending on how difficult the target was to significantly knock down. During the whole siRNA treatment, typically 36–44 h long, 2.5 μM milrinone was included to prevent resumption of meiosis. Next, oocytes were transferred into milrinone-free MEM+ and cultured for 8 or 16 h. They were then used for the phenotype analysis-related experiment described below.

### SiRNA microinjection

For siRNA microinjection, 7 pl of siRNA (5 μM) mixture was injected into each GV oocyte with a IM300 Programmable Microinjector (Narishige, Japan) on Nikon NT-88-V3 Micromanipulation System with heating stage (Nikon, Japan). M2 medium was used to keep stable PH during microinjection. 2.5 μM milrinone was included to prevent resumption of meiosis.

### Immunofluorescence

Oocytes were briefly washed in PBS with 0.05% polyvinylpyrrolidone (PVP), permeated in 0.5% Triton X-100 / PHEM (60 mM PIPES, 25 mM Hepes pH 6.9, 10 mM EGTA, 8 mM MgSO4) for 5 min and washed three times rapidly in PBS / PVP. Next the oocytes were fixed in 3.7% paraformaldehyde (PFA) / PHEM for 20 min, washed three times (10 min each) in PBS / PVP and blocked with blocking buffer (1% BSA / PHEM with 100 mM glycine) at room temperature for 1 h. Then the oocytes were in sequence incubated at 4°C overnight with primary antibody diluted in blocking buffer, washed three times (10 min each) in PBS with 0.05% tween-20 (PBST), incubated at room temperature for 45 min with secondary antibody diluted in blocking buffer (1:750 in all cases), washed three times (10 min each) in PBST. Finally DNA was stained by 10 μg / ml Hochest 33258 (Sigma) and the oocytes were mounted onto a slide with mounting medium (0.5% propgal gallate, 0.1M Tris-Hcl, PH7.4, 88% Glycerol) and covered with a cover glass (0.13–0.17 μm thick). To maintain the dimension of the oocytes, two strips of double-stick tap (90 μm thick) were sticked between the slide and cover glass. Dilution of primary antibody are as follows: anti-TRIM75, 1:200; anti-tubulin, 1:500; anti-human centromere, 1:500. The oocytes were examined with an Andor Revolution spining disk confocal workstation (Oxford instruments, Belfast, Northern Ireland).

### Western blotting

100 oocytes were collected in SDS sample buffer (Biorad) and boiled for 4–5 min, then cooled on ice and centrifuged at 14000 rpm for 5 min to remove pellets. The proteins were separated by sodium dodecyl sulfate-polyacrylamide gel electrophoresis (SDS-PAGE) with a 4% stacking gel and a 10% separating gel for 1.5 h at 100 voltage (V) and then electrophoretically transferred onto polyvinylidene fluoride (PVDF) membrane for 1.5 h at 100 V at 4°C. After being washed three times (10 min each time) in TBS (20 mM Tris, 137 mM NaCl, pH 7.4), the PVDF membrane was blocked in blocking buffer (TBS with 0.04% Tween20 and 2.5% low-fat milk) at room temperature for 1 h. Next, the membrane was in sequence incubated with goat anti-trim75 antibody diluted 1:500 in blocking buffer at 4°C overnight, washed three times (10 min each) in TBS with 0.04% Tween20 (TBST), incubated with HRP-conjugated Rabbit anti-Goat IgG (H+L) diluted 1:5000 in blocking buffer at room temperature for 1 h, washed three times (10 min each) in TBST. Finally signal detection was performed by the enhanced chemiluminescence (ECL) technique using ECL Advance reagents (Amersham Biosciences UK Limited, Little Chalfont Buckinghamshire, England). For actin detection, the dilution for mouse anti-actin antibody is 1:3000, the dilution for HRP-conjugated goat anti-mouse IgG (H+L) dilution is also 1:5000.

### Area and fluorescence intensity measurement

To measure the area of chromosome or spindle, oocyte immunofluorescence image with chromosome and spindle staining is opened with Image J and line is drawed around the edge of chromosome or spindle with "polygon selections" tool and the area of the closed line region are measured with "measure" tool. Fluorescence intensity measurement of cytoplasmic FITC-conjugated siRNA is done the same way, but only the net cytoplasmic intensity, which is obtained by substracting the total cytoplasmic intensity with background intensity (Average intensity of the region beside oocyte cytoplasm), is used for final statistics.

### Data analysis and statistics

All experiments were repeated at least three times, Measurement on confocal Images was done with Image J. Data were presented as average ± Sem. Statistical comparisons were made with Student’s test of EXCEL. P<0.05 was considered to be statistically significant.

## Results and Discussion

### Peptide nanoparticle-mediated siRNA can efficiently enter intact mouse oocytes

**Fig 1:**
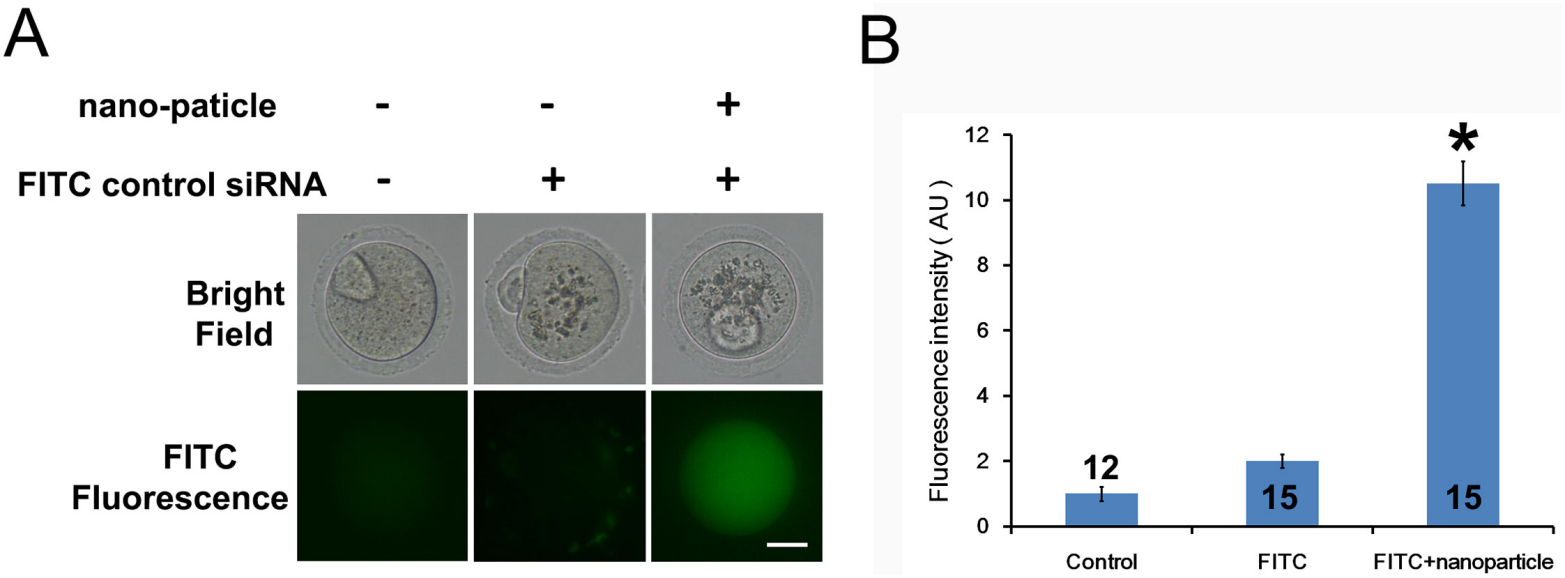
Peptide nanoparticle-mediated siRNA can efficiently enter intact mouse oocytes. (A) Fluorescence comparison between the three different groups. Left, control oocytes without any treatment; middle, oocytes incubated with FITC-conjugated control siRNA only; right, oocytes incubated with peptide nanoparticle-encapsulated FITC-conjugated control siRNA. Corresponding bright field images of live oocytes are shown in the upper panel. GV oocytes were treated and then cultured till MII. (B) Quantification of FITC fluorescence of the three groups in A. Numbers in or above columns are numbers of oocytes measured. A significant comparison (p<0.05) is indicated with an asterisk (*). Scale bar, 20 μm.

To test whether peptide nanoparticle-mediated siRNA can efficiently enter intact mouse oocytes, we used an fluorescein isothiocyanate (FITC)-conjugated control siRNA and compared the difference in cytoplasmic fluorescence between control oocytes, oocytes incubated with FITC–siRNA only, and oocytes incubated with peptide nanoparticle-complexed FITC–siRNA. We treated the GV oocytes and cultured them till they reached MII stage; subsequently, the FITC fluorescence images of live oocytes were taken and measured. Results (Fig 1A and 1B) showed that the cytoplasmic fluorescence of control oocytes was very low and almost the same as that of oocytes incubated with FITC–siRNA only. Meanwhile, the cytoplasmic fluorescence of oocytes incubated with peptide nanoparticle-complexed FITC–siRNA was significantly higher than those of the other two groups, indicating that peptide nanoparticle-complexed FITC–siRNA had been efficiently delivered into the oocytes. The oocytes from the three groups all looked healthy and developed to the MII stage synchronously (Fig 1A), indicating that the peptide nanoparticle was safe to oocytes.

Several key factors affect the operability of peptide nanoparticle-mediated siRNA transfection in mouse oocytes. First, high-quality FBS with lowest album and endotoxin level can efficiently prevent cell damage caused by peptide nanoparticles. Although the toxicity of peptide nanoparticles is low, they are still somewhat harmful to oocytes because oocytes are very sensitive to all toxins. Therefore, we included 20% Performance-Plus grade Gibco FBS throughout the processes of siRNA transfection (For all other experiments, regular Secure grade Gibco FBS is good enough). Second, FBS has to be included in all media (including the oocyte handling solution) immediately after GV oocytes were removed from their follicles. This is because the zona pellucida hardens quickly without FBS, completely blocking the entry of the peptide nanoparticle–siRNA complex. Third, the amounts of nanoparticles and siRNA have to be optimized to ensure that siRNA is efficiently encapsulated inside the peptide nanoparticles.

### Peptide nanoparticle-mediated siRNA transfection can efficiently and specifically knock down gene expression

**Fig 2:**
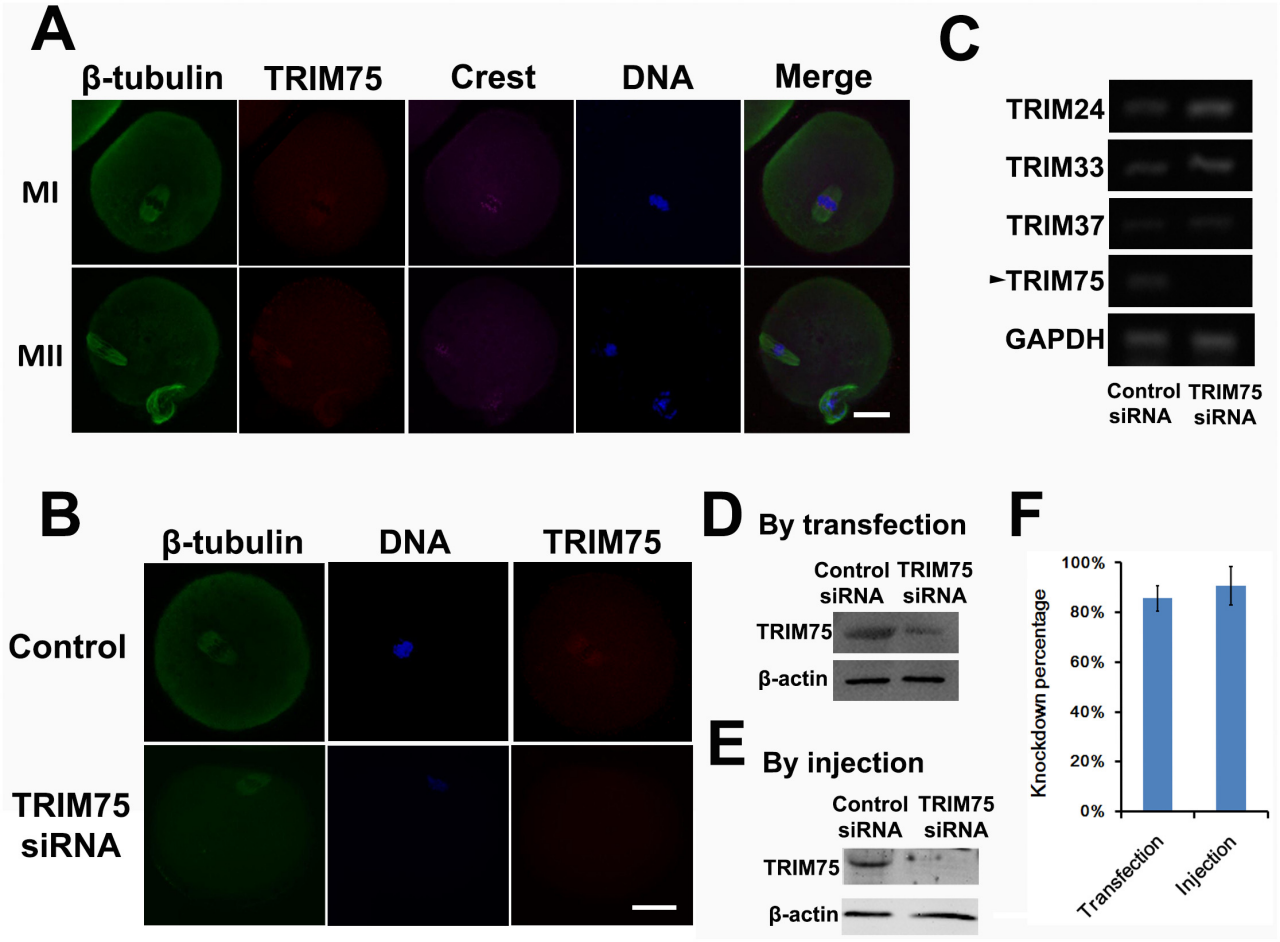
Peptide nanoparticle-mediated siRNA transfection can efficiently and specifically knock down gene expression. (A) Trim75 localizes within spindles in MI (upper) and MII (lower) oocytes. Tubulin is shown in green, trim75 in red, kinetochores in magenta, and DNA in blue. (B) Trim75 staining in control (upper) and TRIM75 siRNA-treated MI oocytes. Trim75 staining significantly decreased after trim75 siRNA treatment. (C) RT-PCR showed that TRIM75 mRNA level significantly decreased after TRIM75 siRNA treatment (arrow), whereas the mRNA levels of the other TRIM family members, TRIM24, TRIM33, and TRIM37, did not change. GAPDH was used as a control. (D) Western blot showed that trim75 significantly decreased after TRIM75 siRNA transfection. β-actin was used as a control. (E) Western blot showed that trim75 also significantly decreased after TRIM75 siRNA microinjection. β-actin was used as a control. (F) Blot quantification showed that trim75 knockdown by transfection is as good as by microinjection. Scale bar, 20 μm.

To test whether nanoparticle-mediated siRNA can efficiently knock down gene expression, we used an siRNA mixture corresponding to four CDS regions of TRIM75 (Table 1). TRIM (tripartite motif-containing) 75 was selected as a female fertility factor in a genome-wide mRNA screen in a fox3-overexpression mouse model. It is expressed predominantly in oocytes compared with ovarian somatic cells or cumulus cells [12], but till now there has been no research into its role in meiosis (male or female) or mitosis. Immunofluorescence showed that trim75 localized within the spindle (Fig 2A), and siRNA significantly reduced trim75 staining within the spindle (Fig 2B). RT-PCR and western blot also showed that the siRNA mixture reduced TRIM75 mRNA and protein levels to less than 20% (Fig 2C and 2D). To test whether the knockdown may affect other members of trim families, we simultaneously tested the mRNA levels of TRIM24, TRIM33, and TRIM37, along with TRIM75. RT-PCR showed that TRIM75 siRNA mixture did not affect the mRNA levels of other TRIMs (Fig 2C). This result indicated that peptide nanoparticle-mediated siRNA transfection can efficiently and specifically knock down gene expression. To directly compare this knockdown method with traditional microinjection, we also microinjected TRIM75 siRNA mixture into mouse oocytes and check the trim75 protein level. We found that our method could knock down as well as microinjection (Fig 2E and 2F).

To the best of our knowledge, this is the first study to show that peptide nanoparticle-mediated siRNA transfection can efficiently enter intact oocytes and knock down specific genes without affecting other members of the same family. The peptide nanoparticle we used in our study was a fusion peptide of HIV-1 gp41 protein and the nuclear localization sequence (NLS) of SV40 large T antigen. It forms stable non-covalent complexes with nucleic acids and improves their delivery [11–12]. We also tried several liposome-based siRNA transfection reagents on mouse oocytes, but we were unable to collect data as massive oocyte death (70%–100%) always occurred within 24 h of transfection. For a long time, the study of meiosis in oocytes has been slow to progress because of several disadvantages of technique compared with mitosis studies; the major one is that, to study a functionally unknown gene, microinjection of specific siRNA into GV oocytes is usually the first key step [7–8]. Certainly, an alternative is to generate knockout mice; however, compared with a simple transfection followed by phenotype analysis within 3 days, the generation of knockout mice is far too time-consuming. Particularly, if we initially want to screen the function of hundreds of candidates potentially involved in certain cellular process, the generation of hundreds lines of knockout mice appears too costly and time-consuming. In mitosis, multiple genome-wide studies employing siRNA-based gene silencing have been performed to uncover new candidates involved in certain important processes [1–3]. However, a similar study in meiosis using microinjection could cost substantially more time and labor [13]. Therefore, we propose that peptide nanoparticle-mediated siRNA transfection should be an alternative potent tool for genome-wide functional screening in female meiosis.

### Effective peptide nanoparticle-mediated siRNA silencing can be used in gene function analysis

**Fig 3:**
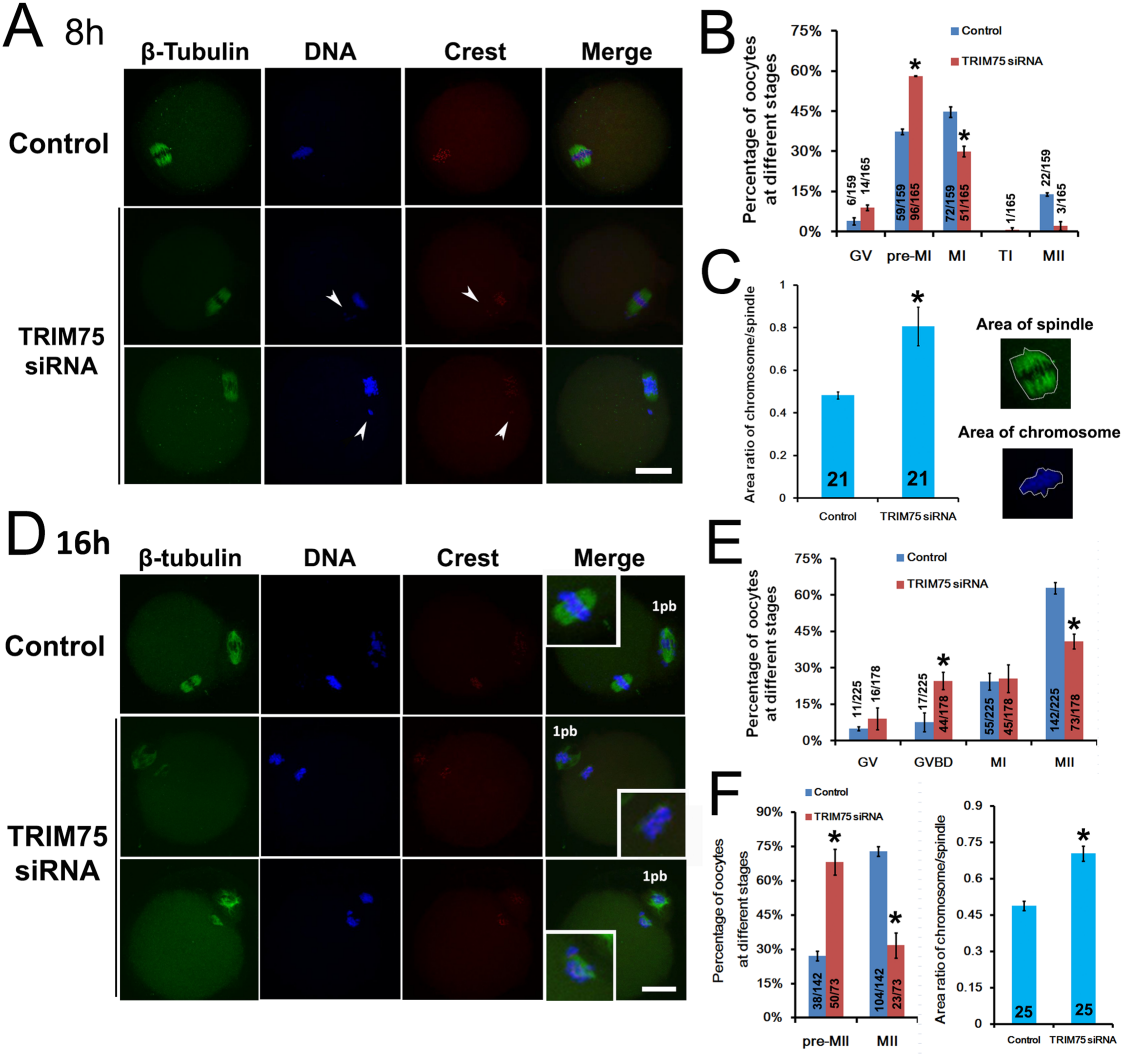
Effective peptide nanoparticle-mediated siRNA silencing can be used in gene function analysis. TRIM75 was knocked down by peptide nanoparticle-mediated siRNA in GV oocytes after which the oocytes were cultured for 8 h or 16 h. Next, the oocytes were fixed for immunostaining and subsequent meiotic phenotype analysis. Integers in columns showed how many oocytes were measured, fractions in or above columns showed number of oocytes at certain stage / number of all examined oocytes. (A) and (B) At 8 h, there were significantly more pre-MI and less MI oocytes in the TRIM75 siRNA group than in the control. (C) At 8 h, chromosomes of pre-MI oocytes in the TRIM75 siRNA group were significantly less congressed than those in the control, as shown by the area ratio of chromosome:spindle. Diagram on the right shows how to determine the area of chromosome or spindle. (D) and (E) At 16 h, the percentage of total MII oocytes was significantly lower in the TRIM75 siRNA group than in the control. (F) At 16 h, there were significantly more pre-MII and less normal MII oocytes in the TRIM75 siRNA group than in the control. (G) At 16 h, chromosomes of pre-MII oocytes in the TRIM75 siRNA group were significantly less congressed than those in the control, as shown by the area ratio of chromosome:spindle. Significant comparisons (p<0.05) are indicated with an asterisk (*).

To test whether peptide nanoparticle-mediated siRNA silencing can be a powerful tool to study the function of unknown proteins, we analyzed the meiotic phenotype after siRNA knockdown of TRIM75. Trim75 contains four domains: B-Box-type zinc finger and RING-finger (Really Interesting New Gene) domain are involved in protein-protein interaction, PRY/SPRY domain and tripartite motif domain are mostly found in novel regulators of immune responses. Although trim75 has not been studied in association with mitosis or meiosis, some family members with similar domain constitution have been closely linked. For example, trim36 has a ubiquitin ligase activity, interacts with centromere protein-H, and colocalizes with alpha-tubulin; excess TRIM36 may cause chromosomal instability [14]. Trim22 (Staf50), an interferon-inducible protein as well as a p53 target gene, is suggested to play a role in viral defense by restriction of viral replication. Trim22 localizes to centrosomes independent of microtubules [15]. RNF33/TRIM60 interacts with kinesin motors KIF3A–KIF3B and possibly contributes to kinesin-dependent mobilization of specific cargo(s) along microtubules in mouse testis [16]. Because trim75 mainly localizes within spindles, we suggest that it might function in spindle organization and thereby affect meiosis. Therefore, we performed a systematic phenotypic analysis on meiotic spindles at 8 or 16 h of IVM. At 8 h, there were significantly more oocytes at pre-MI (control vs TRIM75, 37.4% vs 58.1%) and accordingly less oocytes at MI (control vs TRIM75, 44.7% vs 30%) in the TRIM75 siRNA group than that in the control siRNA group (Fig 3A and 3B). Moreover, chromosomes in pre-MI oocytes in the trim75 siRNA group were significantly less congressed than those in control groups as revealed by the area ratio of DNA:spindle (Fig 3C, control vs TRIM75, 0.48 vs 0.81). At 16 h, there were significantly less oocytes developed to MII stage (Fig 3D and 3E, control vs TRIM75, 62.9% vs 40.9%). Moreover, significantly more MII oocytes in the TRIM75 siRNA group had uncongressed chromosomes and disorganized spindles; we called these oocytes “Pre-MII” (Fig 3D and 3F, percentage of Pre-MII oocytes, control vs TRIM75, 27.1% vs 68.3%; Fig 3D and 3G, area ratio of DNA/spindle, control vs TRIM75, 0.49 vs 0.70). These results indicated that trim75 is important for female meiosis, and peptide nanoparticle-mediated siRNA knockdown can be an efficient alternative tool to study the function of unknown proteins in mouse oocytes.

## Conclusions

For the first time, we have successfully developed peptide nanoparticle-mediated siRNA transfection for efficient gene knockdown in mouse oocytes and showed that it can be used in the functional study of unknown genes. We hope this new tool will contribute to the investigation of female meiosis.
